# Correction: Outer hair cell electromotility is low-pass filtered relative to the molecular conformational changes that produce nonlinear capacitance

**DOI:** 10.1085/jgp.20181228012112023c

**Published:** 2023-12-22

**Authors:** Joseph Santos-Sacchi, Kuni H. Iwasa, Winston Tan

Vol. 151, No. 12 | https://doi.org/10.1085/jgp.201812280 | November 1, 2019

In the Appendix of this paper, two transition rates, *k*_+_ and k_−_, did not include the effect of the energy barrier between the two conformational states. The inclusion of this barrier effect required redefinition of those two transition rates. In addition, there were typographical errors in Eqs. 38 and 41, which have been corrected. The corrections appear in bold below. In addition, the values on the x axis in Fig. A3 were changed to kilohertz. These errors were fixed in the online article but appear in print and in the PDF.

## Motile element with two states

Consider a membrane molecule with two discrete conformational states, C_0_ and C_1_, and let the transition rates *k*_+_ and *k*_−_ between them be schematically expressed as

$$C0\;\begin{array}{c} k_{+}\\ ↔\\ k_{-} \end{array}C1.$$

Let *P*_1_ be the probability that the molecule in state C_1_. Then, the probability *P*_1_ can be expressed by the transition rates

$$\frac{P_{1}}{1-P_{1}}=\frac{k_{+}}{k_{-}}=\exp\left\{-\beta \left[q\left(V-V_{1/2}\right)\right]\right\},$$ (A1)

where *q* is the charge transferred across the membrane during conformational changes, *V* the membrane potential, ***V***_**1/2**_** the half-point voltage of the transition**, and *β* = 1/*k*_B_*T* with the Boltzmann constant and *T* the temperature.

If *q* is positive, the energy level of the state C_1_ is higher, reducing *P*_1_ as the membrane potential *V* rises. For prestin in outer hair cells, which shorten on depolarization, if we choose C_1_ as the shortened state, the unit length change *a* on conformational change is negative, and then we have *q* < 0. Notice that the quantity a does not appear in Eq. A1.

The transition rates that satisfy Eq. A1 can be given by

$$k_{+}\;=\;{\bar{k}}_{+}\exp\left[-\alpha \beta q\left(V\;-\;V_{0}\right)\right]\approx \;{\bar{k}}_{+}\left[1\;-\;\alpha \beta q\left(V\;-\;V_{0}\right)\right],$$ (A2)

$$k_{-}\;=\;{\bar{k}}_{-}\exp\left[\left(1\;-\;\alpha \right)\beta q\left(V\;-\;V_{0}\right)\right]\approx \;{\bar{k}}_{-}\left[1\;+\;\left(1\;-\;\alpha \right)\beta q\left(V\;-\;V_{0}\right)\right].$$ (A3)

**Here, *α* is a constant between 0 and 1 and** ${\bar{k}}_{+}$ **and** ${\bar{k}}_{-}$ **are transition rates at the operating voltage *V***_**0**_ **, around which *V* changes with time. The exponential function can be linearized because we assume *V* − *V***_**0**_ **is small. These rates at the operation point are expressed by**

$${\bar{k}}_{+}\;=\;\bar{k}\exp\left[1\;\;-\alpha \beta q\left(V_{0}\;-\;V_{1/2}\right)\right],$$

$${\bar{k}}_{-}\;=\;\bar{k}\exp\left[\left(1\;-\;\alpha \right)\beta q\left(V_{0}\;-\;V_{1/2}\right)\right],$$

**where the transition rate** $\bar{k}$ **is due to an energy barrier between the two states, excluding the difference in the energy levels, which are voltage dependent.**

The time dependence of *P*_1_ can be expressed by the rate equation

$$\;\frac{d}{dt}P_{1}=k_{+}-\left(k_{+}+k_{-}\right)P_{1}.$$ (A4)

Now we introduce sinusoidal voltage changes of small amplitude v on top of constant voltage $\bar{V}$, i.e., $V=\bar{V}+v\;\exp\left[i\omega t\right]$, where *ω* is the angular frequency and i = √−1. Then the transition rates are time-dependent due to the voltage dependence Eq. A1. They satisfy

$$\frac{k_{+}}{k_{-}}=\frac{{\bar{k}}_{+}}{k_{-}}\left(1-\beta q\nu \;\exp\left[i\omega t\right]\right).$$ (A5)

Notice ${\bar{k}}_{-}$ and ${\bar{k}}_{+}$ are time independent, and we assume that *v* is small so that *βqv* << 1. A set of *k*_+_ and *k*_−_ that satisfies Eq. A5 can be expressed

$$k_{+}\;=\;{\bar{k}}_{+}\left(1\;-\;\alpha \beta q\nu \exp\left[i\omega t\right]\right),$$ (A6)

$$k_{-}\;=\;{\bar{k}}_{-}\left\{1\;-\left(1-\alpha \right)\;\beta q\nu \exp\left[i\omega t\right]\right\}.$$ (A7)

If we express $P_{1}={\bar{P}}_{1}+p_{1}\exp\left[i\omega t\right]$, we have respectively for the 0th and first order terms (Iwasa, 1997)

$${\bar{P}}_{1}=\frac{{\bar{k}}_{+}}{k_{+}+k_{-}},$$ (A8)

$$p_{1}=-\frac{{\bar{k}}_{+}{\bar{\bar{k}}}_{-}}{{\bar{k}}_{+}+{\bar{\bar{k}}}_{-}}\cdot \frac{\beta q\nu }{i\omega +{\bar{k}}_{+}+{\bar{k}}_{-}}.$$ (A9)

Notice that *p*_1_ does not depend on the factor *α*.

Eq. A9 leads to voltage-driven mechanical displacement *ap*_1_ exp[*iωt*] with

$${ap}_{1}={-\bar{P}}_{\pm }\pm \cdot \frac{\beta aqv}{1+i\omega /\omega_{g}},$$ (A10)

where ${\bar{P}}_{\pm }={\bar{P}}_{1}\left(1-{\bar{P}}_{1}\right)$. The amplitude |*χ*| of the motile response is given by

$${\left|x\right|}^{2}=\frac{{\left(\beta aq{\bar{P}}_{\pm }\right)}^{2}}{1+{\left(\omega /\omega_{g}\right)}^{2}}\cdot v^{2}.$$ (A11)

Charge displacement is expressed by *qp*_1_ and the contribution to complex admittance *Y*(*ω*) is given by (*q*/*v*)(*d*/*dt*)*p*_1_ exp[*iωt*] (Iwasa, 1997). The contribution to the membrane capacitance is *C*_nl_(*ω*) = *Im*[*Y*(*ω*)]/*ω* and therefore

$$C_{nl}\left(\omega \right)=\frac{\beta q^{2}{\bar{P}}_{1}\left({\bar{P}}_{1}\right)}{1+{\left(\omega /\omega_{g}\right)}^{2}}.$$ (A12)

This contribution to the membrane capacitance is commonly referred to as NLC because it shows marked voltage dependence. Notice also that the above derivation evaluates the contribution of a single unit of motile element. For a cell that contains *N* motile units, both |*x*| and *C*_nl_ need to be multiplied by *N*.

The roll-off frequency *ω*_g_ due to gating is expressed by

$$\omega_{g}\;=\;{\bar{k}}_{+}\;+\;{\bar{k}}_{-}.$$ (A13)

**With Eqs. A6 and A7**, this means that 1/*ω*_g_ rises at both ends of the membrane potential because *α* can take any value between 0 and 1. That means *ω*_r_ can be asymmetric unless *α* = 1/2.

In the special case of *α* = 1/2, ${\bar{k}}_{+}$ = 1/${\bar{k}}_{-\;}$. If we define $b\left(V\right)=\exp\left[-\beta q\left(\bar{V}-V_{0}\right)/2\right]$, then

$$\omega_{g}\;=\;{\bar{k}}_{+}b\left(V\right)\;+\;{\bar{k}}_{-\;}/b\left(V\right),$$ (A14)

which resembles the bell-shaped voltage dependence of nonlinear capacitance at low frequencies (*ω* → 0).

## Mechanoelastic coupling

For motile membrane proteins based on mechanoelectric coupling, charge transfer is affected by mechanical factors. Here, we assume the cell is cylindrical as in the case of cochlear outer hair cells and approximate it as a one-dimensional object (Fig. A1).

Supposing charge transfer *q* is associated with a change *a* in the length of the cell, Eq. A1 should be replaced by

$$\frac{P_{1}}{1-P_{1}}=\frac{k_{+}}{k_{-}}=\exp\left\{-\beta \left[q\left(V-V_{1/2}\right)+aF\right]\right\},$$ (A15)

where ***V***_**1/2**_** is the midpoint voltage of the Boltzmann function and ***F* is the axial force. **Eq. A16 and A17 for the transition rates should be**

$$k_{+}\;=\;{\bar{k}}_{+}\exp\left[-\alpha \beta \left[q\left(V\;-V_{0}\right)\;+\;af\right]\right],$$ (A16)

$$k_{-}\;=\;{\bar{k}}_{-}\exp\left[\left(1\;-\;\alpha \right)\beta \left[q\left(V\;-\;V_{0}\right)\;+\;af\right]\right],$$ (A17)

**where *f* is a small change in the axial force *F* that corresponds to a small voltage change *V*** − ***V***_**0**_**. The transition rates **${\bar{k}}_{+}$** and **${\bar{k}}_{-}$** are redefined by including the effect of the axial force *F***_**0**_** of the resting condition**. For the rest of the present paper, the dependence on the value of the parameter *α* does not appear except for *ω*_g_.

With a shorthand notation ${\bar{P}}_{\pm }\left(={\bar{P}}_{1}\left(1-{\bar{P}}_{1}\right)\right)$, the change of the conformational probability *p*_1_ can be driven either by changes in the voltage as well as force:

$$p_{1}=-\beta {\bar{P}}_{\pm }\cdot \frac{qv+af}{1+i\omega /\omega_{g}}.$$ (A18)

If the motile element is driven by voltage changes, *p*_1_ is proportional to *v* and mechanical displacement is given by *ap*_1_.

## Effect of viscous drag

Movement is driven by a deviation from Boltzmann distribution. When voltage changes with amplitude *v* is imposed, *p*_1_ as expressed by Eq. A18 is the goal of the drive. Since this force is countered by viscous drag (with drag coefficient *η*), the equation of motion in the frequency domain can be expressed by

$$i\eta \omega ap\;=\;ka\left(p_{1}-\;p\right).$$ (A19)

Notice here that the equilibrium transition rates here depend not only on $\bar{V}$ but also on $\bar{F}$ because the motile element based on piezoelectricity is sensitive to mechanical force as well as the membrane potential.

Eq. A19 leads to

$$\left(1+i\omega /\omega_{\eta }\right)p=-\frac{\beta {\bar{P}}_{\pm }}{1+i\omega /\omega_{g}}\cdot qv,$$ (A20)

similar to the previous treatment for the special case of without inertial loading (Iwasa, 2016). Here, the viscoelastic relaxation frequency is defined by *ω*_*η*_ = *k*/η. It is essentially an equation for viscoelastic relaxation, adding a low pass filter to the motile mechanism. It is consistent with previous expressions in both extremes, i.e., *ω*_g_ → ∞ and *ω*_*η*_ → ∞.

The voltage dependence of NLC and that of motile response are identical. In the following, we show that mechanical load with complex relaxation can lead to discrepancy in their frequency dependences.

## Complex mechanical relaxation

Let *X* represent the point that links a spring *k*_1_ with a dashpot *η*_1_. Let *Y* represent the point that joins the spring *k*_1_ with the rest, which includes a spring *k*_2_, a dashpot *η*_2_, and a driver (Fig. A2). The equations of motion of this system driven by force *F* generated at the location *P* can be expressed

$$\eta_{1}\frac{dX}{dt}=-k_{1}\left(X-Y\right),$$ (A21)

$$F+k_{2}Y+\eta_{2}\frac{dY}{dt}=k_{1}\left(X-Y\right).$$ (A22)

If the force generator operates at a frequency *ω* with small amplitude on top of its steady value $\bar{F}$,F = $\bar{F}$ + *f* exp[*iωt*]. By letting the small amplitude components of *X* and *Y* with frequency *ω* represented respectively by *x* and *y*, Eqs. A21 and A22 turn into

$$i\omega \eta_{1}x=-k_{1}\left(x-y\right),$$ (A23)

$$f+\left(k_{2}+i\omega \eta_{2}\right)y=k_{1}\left(x-y\right).$$ (A24)

Eq. A23 can be rewritten as

$$x=\frac{y}{1+i\omega /\omega_{1}},$$ (A25)

which indicates that the quantity *x* is obtained by low-pass filtering ***y*** with roll-off frequency of *ω*_1_(= *k*_1_/*η*_1_).

By introducing a characteristic frequency *ω*_2_(= (*k*_1_ + *k*_2_)/*η*_2_), Eq. A24 can be transformed into

$$f+\left(k_{1}+k_{2}\right)\left[1+i\omega /\omega_{2}\right]y=k_{1}x.$$ (A26)

Elimination of *x* from Eq. A26 with the aid of Eq. A25 leads to

$$y=f/G_{1}\left(\omega \right),$$ (A27)

$$G_{1}\left(\omega \right)=\frac{k_{1}}{1+i\omega /\omega_{1}}-\left(k_{1}+k_{2}\right)\left(1+i\omega /\omega_{2}\right).$$ (A28)

An approach analogous to those in the previous sections lead to an equation

$$G_{1}\left(\omega \right)ap=ak_{2}\left(p_{1}-p\right).$$ (A29)

Since we have *y* = *ap*, this equation leads to

$$\left[G_{1}\left(\omega \right)+k_{2}\right]y=-\frac{\beta {\bar{P}}_{\pm }k_{2}aqv}{1+i\omega /\omega_{g}}.$$ (A30)

Eqs. A25 and A30 show that the relationship between *y* and *v* has three adjustable parameters, *ω*_1_, *ω*_2_, and *k*_*r*_ (= *k*_1_/*k*_2_). For an example of the frequency dependence of *y*, see Fig. A3.

The frequency dependence of NLC is the same as that of *y*. Motile response *x* is obtained by low-pass filtering *y*. The roll-off frequency of *y* is voltage-dependent due to the voltage dependence of *ω*_g_.

With the connectivity of Fig. A2, it is difficult to make high frequency roll off of both quantities as similar as the experimental data. For *y* to roll off at relatively high frequency, *ω*_1_ has to be small and *ω*_2_ has to be large because *G*_1_ must be small as required by Eq. A30. This requirement makes *x* roll off at a frequency much lower than *y* does (Fig. A3).

## Modified complex mechanical relaxation

The model described above predicts a difference between *x* and *y* much larger than the experimentally observed frequency dependence. Let us add a spring across the upper dashpot (Fig. A4).

The set of equations that describe this configuration are

$$\left(\eta_{0}\frac{d}{dt}+k_{0}\right)X=k_{1}\left(Y-X\right),$$ (A31)

$$F+\left(\eta_{2}\frac{d}{dt}+k_{2}\right)Y=-k_{1}\left(Y-X\right).$$ (A32)

If force *F* is driven at angular frequency *ω* with amplitude *f*, the equation is transformed into

$$\left(i\omega \eta_{0}+k_{0}\right)x=k_{1}\left(y-x\right),$$ (A33)

$$f+\left(i\omega \eta_{2}+k_{2}\right)y=-k_{1}\left(y-x\right),$$ (A34)

where variables in the lower case *x* and *y* are the complex amplitude of frequency *ω*.

Eqs. A33 and A34 can be rewritten as

$$x=\frac{k_{1}}{k_{0}+k_{1}+i\omega \eta_{0}}\cdot y,$$ (A35)

$$f=k_{1}x-\left(k_{1}+k_{2}+i\omega \eta_{2}\right)y.$$ (A36)

In the manner similar to Eq. A27 and A28 in the previous case, these equations can be expressed as

$$y=f/G_{2}\left(\omega \right),$$ (A37)

$$G_{2}\left(\omega \right)=\frac{{k_{1}}^{2}}{k_{0}+k_{1}+i\omega \eta_{0}}-\left(k_{1}+k_{2}+i\omega \eta_{2}\right),$$ (A38)

which corresponds to *G*_1_(*ω*) in the previous case.

Since force generation is associated with spring ***k***_**2**_ in the manner similar to the previous case, we obtain

$$\left[G_{2}\left(\omega \right)+k_{2}\right]y=-\frac{\beta aqk_{2}{\bar{P}}_{\pm }}{1+\frac{i\omega }{\omega_{g}}}\cdot v,$$ (A39)

and *x* is obtained with Eq. A35.

If the cell contains *N* motile units, *a* should be replaced by *aN*. For numerical analysis, the number of parameters can be reduced by introducing the ratios *k*_02_(= *k*_0_/*k*_2_), *k*_12_(= *k*_1_/*k*_2_), *ω*_0_(= *k*_2_/*η*_0_), and *ω*_2_(= *k*_2_/*η*_2_), and *x* and *y* are expressed by

$$x=\frac{k_{12}}{k_{02}+k_{12}+i\omega /\omega_{0}}\cdot y,$$ (A40)

$$y=\frac{\beta aqN{\bar{P}}_{\pm }}{1+i\omega /\omega_{g}}\cdot \frac{1}{k_{12}^{2}/\left(k_{02}+k_{12}+i\omega /\omega_{0}\right)-\left(k_{12}+i\omega /\omega_{2}\right)}\cdot v.$$ (A41)

The corresponding equations (Eq. 10) in the main text are expressed with linear frequency *f* instead of angular frequency *ω*. Because these equations depend only on frequency ratios, no extra factor appears.

**Figure A3:**
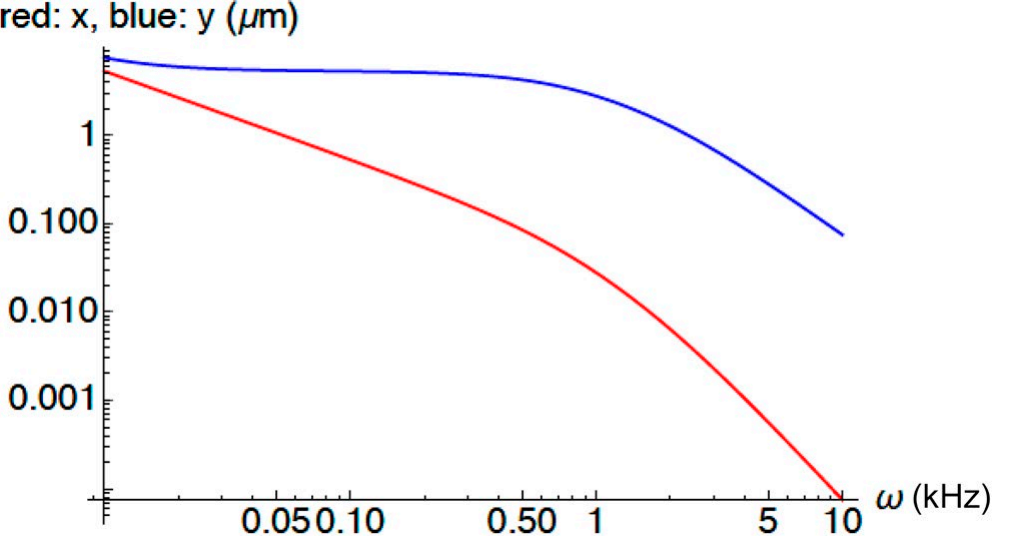
**An example of the frequency dependence of *x* (red) and *y* (blue).** Parameter values: *ω*_g_ = 2, *ω*_1_ = 0.01, *ω*_2_ = 5.12, and $\frac{k_{1}}{k_{2}}$ = 0.16.

